# A Critical Review of the Social and Behavioral Contributions to the Overdose Epidemic

**DOI:** 10.1146/annurev-publhealth-090419-102727

**Published:** 2021-11-30

**Authors:** Magdalena Cerdá, Noa Krawczyk, Leah Hamilton, Kara E. Rudolph, Samuel R. Friedman, Katherine M. Keyes

**Affiliations:** 1 Center for Opioid Epidemiology and Policy, Department of Population Health, Grossman School of Medicine, New York University, New York, NY 10016, USA; 2 Department of Epidemiology, Mailman School of Public Health, Columbia University, New York, NY 10027, USA

**Keywords:** opioids, overdose, supply, demand, policy, social determinants

## Abstract

More than 750,000 people in the United States died from an overdose between 1999 and 2018; two-thirds of those deaths involved an opioid. In this review, we present trends in opioid overdose rates during this period and discuss how the proliferation of opioid prescribing to treat chronic pain, changes in the heroin and illegally manufactured opioid synthetics markets, and social factors, including deindustrialization and concentrated poverty, contributed to the rise of the overdose epidemic. We also examine how current policies implemented to address the overdose epidemic may have contributed to reducing prescription opioid overdoses but increased overdoses involving illegal opioids. Finally, we identify new directions for research to understand the causes and solutions to this critical public health problem, including research on heterogeneous policy effects across social groups, effective approaches to reduce overdoses of illegal opioids, and the role of social contexts in shaping policy implementation and impact.

## NATIONAL TRENDS IN OPIOID USE AND RELATED HARMS

Opioid use disorder (OUD) is a complex condition with biological, behavioral, and social causes. It is associated with substantial morbidity and mortality, including overdose, increased risk of blood-borne infections through injection, and pregnancy and birth complications, among other concerns (62, 123, 165). Opioid overdose has increased rapidly in many countries, including in the United States in the past two decades. Almost 47,000 persons in the United States died of a drug overdose involving opioids in 2018 (163), a ninefold increase over the 1999 level (66, 136).

The beginning of the opioid overdose epidemic was marked by a rise in prescription opioid (PO) overdose deaths (65). Heroin use and related harms increased in the mid-2000s, surpassing POs as a cause of opioid-related overdose in 2015. The rate of heroin-related overdose increased from 1.0 per 100,000 population in 2010 to 4.9 per 100,000 in 2018. The overdose epidemic escalated again after 2013, when high-potency synthetic opioids such as fentanyl, adulterated heroin, and other illegal drugs increased the lethality of use and the opioid overdose death rate (33). Overdose involving synthetic opioids other than methadone became the predominant opioid cause of death, responsible for 31,335 US deaths in 2018 (163).

Overdose deaths involving stimulants such as cocaine and methamphetamine began to increase rapidly in 2015 (65, 79), due in large part to adulteration of those substances with synthetic opioids. The impact of coronavirus disease 2019 (COVID-19) on overdose death rates remains to be determined, but the increase in social isolation, coupled with reduced access to harm reduction services (12), portends further increases in overdose deaths.

### Differences in the Distribution of Opioid-Related Harms by Race/Ethnicity

The increased rates of opioid-related harms since the early 1990s have differed by race/ethnicity. Prior to the 1990s, OUD was primarily driven by heroin use, was concentrated in densely urban areas with a greater burden among men, and showed few racial/ethnic differences (30). As POs became more prominent in the 1990s and 2000s, increases in PO misuse and overdose deaths were concentrated among non-Hispanic Whites, although American Indian/Alaska Natives experienced substantial increases as well (77).

There is limited evidence for an increase in opioid overdose mortality among African Americans during this period. African Americans are less likely than Whites to be treated with an opioid analgesic for the same conditions and same level of pain, which is indicative of systematic bias in the health care system (145). This undertreatment and lack of attention to equity in the health care system may explain to some extent the lower rates of OUD and overdose among African Americans compared with Whites during the 1990s and 2000s (119). Further, many African American urban communities were disproportionately affected by the HIV/AIDS epidemic and hyperpolicing associated with crack/cocaine. As a result of this, some evidence suggests that the cohort of youth in the 1990s actively avoided opioids, crack, and cocaine, as a reaction to the devastation they had witnessed among older community members (50, 56, 116).

The increase in rates of heroin use and heroin use disorder in the 2010s was greater among Whites compared with other racial/ethnic groups. While rates of overdose from heroin and then synthetic opioids have remained higher among Whites compared with other ethnicities, the largest relative increase in overdose rates in this period occurred among African Americans and American Indian/Alaska Natives (1, 99, 134, 140).

### Geographic Patterns of Misuse and Overdose Across the Country

Although opioid misuse and overdose have been historically concentrated in US urban areas with well-developed drug distribution chains, geographic distributions have shifted. Opioid-related deaths increased the most in states with large rural areas and populations disproportionately affected by deindustrialization, including Kentucky, West Virginia, and Ohio. However, all states evidenced increases in PO-related harm regardless of rurality (82).

As the opioid epidemic grew and shifted toward heroin as a driver of opioid-related death, so did the geographic distribution of opioid-related harms. While large rural areas in Appalachia and outlying regions continued to exhibit the highest burden of opioid-related harm, the rate of increase of heroin-related overdose deaths accelerated in the Midwest and Northeast of the United States (149), including both urban and suburban metro areas (144). Most recently, a wave of fentanyl deaths has affected most areas of the United States but has been particularly concentrated on the East Coast and other eastern regions of the country. In contrast, methamphetamine use has been a significant driver of overdoses in the western regions of the United States in the most recent period (65).

## AN OVERVIEW OF SOCIAL AND BEHAVIORAL CONTRIBUTORS TO THE OVERDOSE EPIDEMIC

The social and behavioral roots of the overdose epidemic can be divided into supply and demand drivers for drug use. We use these terms generally to refer to the way opioids are made available through manufacturing and distribution (supply) and to the factors that make certain groups more vulnerable to using and/or becoming addicted to opioids (demand). While many individual-level factors such as genetics, psychiatric disorders, and adverse childhood events are associated with opioid use, in order to identify the social and behavioral drivers of the overdose epidemic we focus our review on risk factors that changed over time.

### Supply Drivers of the Overdose Epidemic

Supply drivers include the proliferation of opioid prescribing to treat chronic pain as well as changes in the heroin and illegally manufactured opioid synthetics markets.

#### The prescription opioid supply: chronic pain and opioid prescribing.

The supply-side roots of the overdose epidemic in the United States lie at the intersection of two social and behavioral forces that, together, led to the proliferation of opioid prescribing in the 1990s, especially for noncancer acute and chronic pain conditions. The first force was a shift in treatment approaches for chronic noncancer pain, including a campaign by professional pain societies and the US Joint Commission, the nation’s largest accrediting body for health care organizations, to consider pain as the “fifth vital sign” (11) and to improve the quality of care for chronic pain. In 1997, the American Pain Society and the American Academy of Pain Medicine released a consensus statement endorsing the use of opioids to treat chronic noncancer pain, arguing that the risk of addiction from opioids was low (4). At the time, the risk of addiction associated with opioid use was not well understood (44, 155).

The second, related force involved the pharmaceutical industry’s concerted efforts to advocate for the long-term use of opioids as a safe, nonaddictive, effective, and humane alternative to treat chronic noncancer pain (27, 87). These marketing efforts accelerated the shift in treatment approaches for chronic noncancer pain. In 1995, for example, Purdue Pharma introduced OxyContin, an extended-release formulation of oxycodone marketed as a nonaddictive and effective medication for the treatment of chronic pain. In 1996–2002, Purdue Pharma provided funds for educational campaigns supporting the use of opioids to treat chronic noncancer pain and supported efforts by pain professional societies, the US Joint Commission, and the Federation of State Medical Boards to advocate for the use of opioids to treat pain (87). The pharmaceutical industry also spent tens of millions of dollars annually to market the use of POs to physicians (58). States responded by enacting a series of Intractable Pain Acts that removed physician sanctions for the use of opioids to treat intractable noncancer pain (135), and the production, distribution, prescription, and use of opioids proliferated. Indeed, the average opioid prescription rate increased from 96 mg per person in 1997 to over 700 mg per person in 2007 (25). The push for the widespread use of POs and the lack of state and federal oversight also led to the creation of so-called pill mills, or pain management clinics that prescribed opioids to patients who had no medical need for them (25, 71, 132). The expansion of PO supply increased opioid-related harm through several pathways, affecting both individuals who initially used opioids as prescribed but developed impaired control over use and individuals who used nonmedically and obtained opioid prescriptions through family, friends, or an illicit diversion source (71).

#### The illegal opioid supply: shifts in heroin and fentanyl markets.

By 2010, when PO overdose deaths began to stabilize (although at high levels), heroin overdose death rates had begun to increase. The PO and heroin overdose epidemics were closely connected: POs and heroin are pharmacologically similar, and in a departure from prior modes of heroin use initiation, in the current overdose epidemic, most persons who use opioids (PWUO) began using POs before using heroin (26, 31). While state restrictions on POs may have increased the risk of transition to heroin use among people with a dependence on POs (26, 31) (as discussed in the section titled Policy and Practice Responses as Modulators of the Epidemic), shifts in the heroin market also likely played an important role. In the mid-1990s, the US supply of South American heroin increased substantially, while the fraction of the market controlled by Asian heroin decreased dramatically (133). Mexican “black tar” heroin dominated the West Coast market, while Colombian heroin dominated the East Coast. The abundant supply, low price, and high purity of the Colombian heroin reduced the price per gram of pure heroin throughout the country and pushed out the Asian market share; competition between Mexican and Colombian heroin also reduced heroin price in areas where both types of heroin were available (133). One study found that the average inflation-adjusted price of pure heroin per gram decreased from $1,368 in 1993 to $688 in 2008, and for every $100 decrease in price per gram of pure heroin, there was a 2.9% increase in the number of heroin overdose hospitalizations (150).

In 2013, the illegal opioid market shifted again with the introduction of fentanyl, often mixed with heroin or sold as counterfeit oxycodone and benzodiazepines. Fentanyl is cheaper to produce—the wholesale price is one-tenth of heroin’s price by weight—and is 30–40 times stronger than heroin, so that a comparable dose would be 1/300–1/400 of the wholesale price of heroin (102). The potential for profit offered by fentanyl may have contributed to the rise of fentanyl in the illicit opioid market (102) and, soon thereafter, to the escalation of fentanyl-involved opioid and other drug-related deaths in the United States.

### Demand Drivers of the Overdose Epidemic

Demand drivers include, among others, deindustrialization and the resulting limitations to employment opportunities, disability and pain arising from work-related injuries, and the increased concentration of poverty. There is substantial literature on the social determinants of substance use, including stressful life events (e.g., 22), area poverty (e.g., 52), and lack of opportunity (e.g., 164). Perhaps one of the most relevant contributions to framing the demand-side drivers of the opioid epidemic is William Julius Wilson’s work on opportunity structure (164), where he wrote that an absence of an opportunity structure involved “few legitimate employment opportunities, inadequate job information networks, and poor schools” (p. 52) and caused residents to “lack contact or sustained interaction” (p. 64) with individuals in the labor market (164). Though this was written more than 20 years ago, it seems an apt description of some localities that were hit hard by the decline of their local economies—e.g., manufacturing (18) and coal (46). Wilson wrote that living in such a place could result in an effort/reward imbalance, where legitimate efforts to improve one’s situation (effort) were likely to be ineffective (lack of reward) because of “systematic blockage of opportunities in the environment” (164, p. 72).

#### Deindustrialization, un- or underemployment, and other factors related to the lack of opportunity for work.

An extensive literature links economic factors such as those related to a lack of opportunity for work, like unemployment and poverty, with the opioid epidemic, especially for those without a college degree (24, 34, 108, 121). Higher unemployment rates are associated with increased psychological distress, opioid prescribing, opioid misuse, emergency room visits, hospitalizations (68), and overdose mortality (68). The literature linking poor economic opportunity to opioid overdose is generally aligned with research linking poor economic conditions to increased overall rates of illegal drug use (see 117 for a review), overdose emergency department visits (68), and drug overdose deaths (54, 121). A recent study using 2006 data (54) found that a 1% increase in the US county-level unemployment rate was associated with a 4.6% increase in the overdose death rate. Local-level job losses (38), indices of economic distress (113), and declines in housing prices (21) have also been correlated with local-level increases in opioid overdose mortality.

Several quasi-experimental studies provide relatively high-quality evidence that reductions in work opportunities contribute to increases in overdose mortality, including from opioids. Autor et al. (9) found that exogenous shocks in manufacturing labor demand differentially affected working-age males and contributed to this subgroup’s increase in mortality from drug and alcohol overdoses. Pierce & Schott (128) found that implementation of a trade liberalization policy increased mortality due to suicide and drug and alcohol overdose, particularly among White males in counties where manufacturing was the dominant industry. Finally, Venkataramani et al. (153) found that auto plant closures contributed to 8.6 additional opioid overdose deaths per 100,000 individuals over a period of 5 years, assuming the absence of confounding effects over the same period. They also found that the effect was greatest for White males of working age and was only present in the subgroup of counties with the highest share of workers employed in the manufacturing sector (top quintile) (153), which is the subpopulation assumed to be most affected by such closures.

The impact of economic conditions on overdose varies by race/ethnicity, gender, and urbanicity, with White males of working age and working in counties heavily dependent on manufacturing showing the strongest associations between economic conditions and overdoses (10, 21, 128, 138, 153). For example, when looking within age, gender, and race/ethnicity subgroups, Rudolph et al. (138) found that labor force participation was associated with fatal drug overdose for White working-age adults but not for Black or Hispanic/Latino adults. The association of unemployment with drug overdose was stronger for men than for women, and the economic opportunity of one subgroup relative to the others was also associated with fatal drug overdose rates for men but not for women (138). One study found evidence that economic conditions may be more strongly related to drug overdose in urban areas (126), even though rates of overdose may be higher in nonurban areas (137, 146). Much of this research on economic conditions and overdose focused on earlier phases of the overdose epidemic, when White adults experienced the highest overdose risk. Rapid shifts in the relative racial/ethnic distributions of overdose deaths in recent years suggest that the ways economic conditions affect overdose risk may have changed; this is a priority for future study.

#### Disability, pain, and work injury.

In addition to demand-side drivers stemming from a lack of work opportunity, there is another set of factors that may be considered a product of manual, physical jobs where worker health may be underprioritized. Such factors include work injury, pain, and disability. Manual laborers, such as those working in construction, are at particularly high risk of dying from an opioid overdose (122). A national study of construction and extraction workers in 2005–2014 found that they were more likely to report PO misuse than other workers (122). This was partly associated with precarious employment conditions and with missing days of work due to illness or injury. Other occupations at risk of overdose mortality include health care support and food preparation (63, 64).

US surveillance studies documented a rise in disability (i.e., inability to meet self-care needs or carry out other routine tasks) among middle-aged adults (29, 103). Disabled adults, especially middle-aged and lower-income individuals, accounted for the majority of hospitalizations for PO and heroin overdoses during the period 1993–2014 (143). They also likely accounted for one-quarter of PO overdoses in 2008 (109).

The disabled population has been identified as particularly vulnerable to high-risk opioid prescribing practices (129, 151, 154). In fact, there is evidence that pharmaceutical companies targeted the disabled subgroup for POs (152). In a review of disabled Medicare beneficiaries in 2007–2011, nearly 50% of disabled adults had filled at least one opioid prescription, almost 25% had filled at least 6, 8% had four or more opioid prescribers, and 0.3% had been treated for a nonfatal PO overdose (109, 114). Studies among workers’ compensation claimants revealed that opioid use was common shortly after filing a claim and throughout the duration of a claim (23, 57), and misuse was much more common among these individuals compared to those without a disability (157). This cycle may have led to loss of work and subsequent enrollment in Social Security Disability Insurance (SSDI) (55).

The risk of opioid misuse associated with work injury and disability may be partly related to the experience of pain. The rate of pain experienced in the population seems to have increased in the last 20 years, although the subjective component both in self-reporting pain and in seeking medical assistance makes it difficult to be sure of the extent of experienced pain. An analysis by Nahin and colleagues using Medical Expenditure Panel Survey data (118), for example, found that the proportion of adults who reported painful health conditions in the United States increased from 32.9% in 1997/1998 to 41% in 2013/2014. The use of opioids to manage pain more than doubled among those with severe pain in this study.

Disability may also interact with the opioid epidemic in ways external to chronic pain. Disability frequently co-occurs with depression and anxiety (159, 167), which can in turn increase risk for opioid misuse and overdose (28, 49). In addition, disability may create practical barriers to substance use disorder treatment. For example, people with disabilities are less likely to receive medications for OUD (97). Disability may also create barriers to participation in work activities, thereby worsening socioeconomic status (37, 69), social connectedness (69, 162), and emotional well-being (148, 162) and ultimately increasing the risk of nonmedical opioid use (108, 113, 168).

## POLICY AND PRACTICE RESPONSES AS MODULATORS OF THE EPIDEMIC

US states and the federal government have invested billions of dollars implementing laws and policies to address the overdose epidemic. Given the effects they have had on opioid-related harms, such measures should be considered social modulators of the overdose epidemic. We discuss their impact below, classified as supply-side, harm reduction, and demand reduction measures.

### Supply-Side Measures

Supply-side measures include laws regulating access to prescription opioids as well as laws regulating access to illegal opioids.

#### Laws regulating access to prescription opioids.

In response to rising high-risk opioid prescribing and overdose deaths, federal and state governments have enacted guidelines, policies, and laws to regulate opioid prescribing and access to POs. Such measures can be classified into those that restrict legal access to opioids and those that attempt to shape prescribing practices. Measures in the two categories likely jointly contributed to a decline in high-risk opioid prescribing and stabilized PO overdose deaths by 2011, but they may have also contributed to the increase in heroin overdoses starting in 2010.

First, restriction of legal access to opioids took several forms, ranging from laws intended to reduce high-risk opioid prescribing at a population level, such as limitations on the amount or duration that POs can be prescribed or dispensed to patients with acute pain (35), to laws targeting the small fraction of high-risk opioid prescribers, such as pain management clinic laws. Evidence about the impact of mandatory limits on prescribing or dispensing of POs for acute pain is preliminary, as 65% of these laws were enacted in 2017 (35). The available evidence suggests that in the short term, opioid prescribing laws were not associated with declines in PO distribution (36).

Pain management clinic laws target high-volume, high-risk opioid prescribing by imposing operational, personnel, inspection, and other requirements on pain management clinics, including regular inspections, restrictions on cash payments, and civil and criminal penalties when violations occur (105, 139). Some studies, particularly those evaluating the impact of pain management clinic laws in single states, suggest that enactment of these laws contributed to a decline in opioid prescribing and fewer opioid overdose deaths (43, 130). Concerns have been raised about increased demand for heroin and fentanyl provided by rogue pain management clinics following restriction of the PO supply. Several prior studies, which evaluated the short-term impact of these laws in single states on heroin overdose, found no effect (43, 130), potentially because they were conducted in the early years following the enactment of pain management clinic laws, before heroin overdose deaths escalated. Future research should investigate the impact of these laws on heroin and synthetic opioid overdoses since the escalation of heroin overdose deaths in 2010.

Second, measures to shift opioid prescribing practices included the implementation of state prescription drug monitoring programs (PDMPs) and the publication of opioid prescribing guidelines. PDMPs are state-level databases that collect prescribing information when controlled substances such as opioids are dispensed. PDMPs vary widely, but there is some indication that those PDMPs that required prescribers and dispensers to check the PDMP before prescribing, authorized prescribers to access PDMP data, required dispensers to update PDMP data with greater frequency, monitored more drug schedules, and sent unsolicited reports of outlying prescribing and dispensing patterns to prescribers were associated with reduced PO overdose deaths (48). In contrast, with the exception of the most comprehensive PDMPs, PDMP enactment was also associated with an increase in heroin overdose deaths (104).

In 2016, the Centers for Disease Control and Prevention (CDC) released the Guideline for Prescribing Opioids for Chronic Pain, which provided 12 recommendations to primary care clinicians treating adult patients with chronic noncancer pain (42). The guidelines stressed that opioids should only be used when the benefits for pain and function outweighed the risks associated with opioid use, that nonopioid therapy was preferred for treatment of chronic pain, and that if opioids must be used, they should be prescribed at the lowest effective dosage and avoiding concurrent opioids and benzodiazepines when possible (42). A time-series analysis of opioid prescribing before and after publication of the guidelines found that while several opioid prescribing practices had already started to decrease before the guidelines, the time of release of the guidelines was associated with a greater decline in the overall opioid prescribing rate, in the rate of high-dosage prescriptions, and in the rate of overlapping opioid and benzodiazepine prescriptions (19). The impact of the guidelines on opioid overdose has not been examined to date. States and institutions such as Veterans Affairs have also enacted guidelines that specify opioid dosage, length of prescription, and other criteria related to the prescribing of opioids to manage chronic noncancer pain. Their impact on the overdose epidemic is not well understood to date.

At the same time as the state and the federal government were enacting measures to regulate opioid prescribing, and partly in response to them, prescribers’ norms regarding prescribing practices shifted, which likely also contributed to the decline in opioid prescribing. In a review of studies on prescriber attitudes, beliefs, and knowledge about opioid prescribing, Haffajee & French (60) found an increase in the prescribers’ awareness of the overdose epidemic and of policy responses, their awareness of state opioid prescribing guidelines, and their support for clinical interventions to reduce PO misuse.

#### Laws regulating access to illegal opioids: criminalization and its consequences.

Criminalization of opioids is the most widespread policy action used to restrict access to illegal opioids. Criminalizing illegal opioids makes the illegal drug supply more risky and thus forces PWUO to use illegal drugs, which have increased in purity and decreased in price over the years of the overdose epidemic (158). Multiple decades of evidence from the War on Drugs, initiated by the Nixon administration and fortified by the Reagan administration, have demonstrated that criminalizing the supply of illicit drugs does very little to change peoples’ use (15). Instead, criminalization has expanded the carceral state and placed additional public health burdens on the justice system.

The punishment for possession of unprescribed opioids varies substantially between states; however, under federal law, the simple possession of small amounts can carry a fine of up to $1,000 and up to 1 year of incarceration. Under the Controlled Substances Act, people convicted of heroin distribution in any quantity exceeding one kilogram of a substance where heroin is detectable face mandatory minimum prison sentences of 10 years (21 U.S.C. § 841). In recent years, numerous states have also added (or begun prosecuting) drug-induced homicide laws that further criminalize the selling or sharing of drugs when someone has died from overdose (67). While these laws are often described as a way to punish high-level drug traffickers, nearly half of the prosecutions have targeted the family and friends of the victim (14). Furthermore, many people who are prosecuted may also have substance use disorders (95).

Even before the modern rise in opioid use across the United States, severe punishments for illegal opioid possession and distribution led to substantial overrepresentation of PWUO among the prison and jail populations. State jails and prisons reported that 17–19% of their populations frequently used opioids prior to incarceration in 2007–2009 (20). Since that time, we have seen an increase in opioid use (65), which suggests that the opioid-using subpopulation of people incarcerated today is larger than the 2007–2009 estimate. In one state-wide study, 14.8% of deaths after release from prison were opioid related (16), and other studies demonstrated a similarly high opioid involvement and overdose in the deaths of people recently released from jail or prison (93, 110). These findings suggest that the involvement of PWUO in the justice system may increase their risk of overdose mortality.

### Harm Reduction Measures

Harm reduction approaches are designed to meet PWUO in their everyday lives, and they focus on mechanisms to reduce the health and social consequences of substance use (100). These approaches use compassionate and pragmatic ways to help people reduce the health consequences of drug use and improve their quality of life (101). Some widespread approaches to overdose-related harm reduction include Good Samaritan laws, laws regulating naloxone access, and laws encouraging safer use practices. Evidence of their contribution to a potential decline in overdose deaths is promising but still preliminary.

#### Good Samaritan laws.

Good Samaritan laws are legal protections for people who contact first responders about an overdose (and sometimes for the person overdosing), and they were established to reduce barriers to contacting emergency services. As of July 2018, 46 states had established some form of Good Samaritan law (125). These laws provide protection against legal consequences for the possession of illicit drugs or drug paraphernalia; however, their levels of protection vary across states, from the most robust protections against arrest to limited sentencing mitigation. Many states exclude people who are under community supervision from these protections (125).

The population-level impacts of Good Samaritan laws on overdose deaths appear mixed. Some studies found no significant impacts of Good Samaritan laws on fatal overdoses (8, 131), whereas others found decreases in fatal overdoses (106). Smaller-scale survey and qualitative studies showed that many people were not aware that their state had a Good Samaritan law (45, 73), and PWUO were fearful of calling the police for fear of legal consequences even when they lived in states with active Good Samaritan laws (85, 96). Research on if and how different types of Good Samaritan laws have different impacts is needed.

#### Naloxone access laws.

Naloxone is a full agonist medication that can effectively reverse the respiratory and/or central nervous system depression that results from opioid overdose (81). Distributing naloxone, and training both first responders and laypeople in administering it, has been recommended by the World Health Organization as a critical means of reducing overdose fatalities (107). Community-based studies of take-home naloxone administration show consistent effectiveness in reversing overdoses (160). As of 2017, 51 jurisdictions had some form of naloxone access law (124). These laws vary in the ease with which prescribers can distribute naloxone, the legal protections under which they do so, and the legal protections for laypeople who administer naloxone (124).

Population-level analyses of naloxone access laws’ impacts on fatal overdoses show a 9–14% reduction in overdose deaths (106, 131). However, very little is known about how different structures of the naloxone law provisions, and the ways in which these policies are implemented, may impact overdoses. Laypeople’s access to naloxone and training on how to administer this drug are among the potential mechanisms that may influence the effectiveness of these laws. Community studies have shown that people who have been trained to use naloxone are more likely to use it than those who are not trained (73) and that the greater the protections and ease of prescribing for providers, the more the prescriptions of naloxone (53, 166).

Concerns that easing the criminal consequences of possessing drugs and providing an overdose reversal drug for PWUO would lead to an increase in drug use have posed barriers to implementing Good Samaritan and naloxone access laws (41). Although one study found an increase in opioid-related emergency department visits following expanded access to naloxone, this study used only a pre-post design and did not account for the increasing rate of fentanyl in the opioid supply (41). More robustly designed national studies found no increase in opioid misuse associated with either type of law (106, 131).

#### Safer use practices.

Multiple safer use practices show promise for reducing overdoses and other opioid-related harms. Syringe exchanges, which allow for the provision of clean needles to people who use drugs, have substantial support as a mechanism for preventing HIV and hepatitis C virus (7). There are also indications from community studies that participation in syringe exchanges facilitates access to treatment, to naloxone, and to other health services (39, 61, 83). Legal safe consumption, safe injection, and supervised injection sites have yet to be established within the United States; however, international evidence suggests that these facilities reduce overdose deaths (80, 120). Developing legal safe consumption sites could have similar impacts on the US opioid epidemic, should the ongoing efforts to launch these programs in cities like New York and Philadelphia succeed. Unfortunately, the development of safe consumption sites has faced substantial challenges from “not in my backyard” (NIMBY) claims (141), similar to those faced in the establishment of syringe exchanges and drug treatment facilities (86). Finally, with the rise of fentanyl in the illicit opioid supply, tools that help PWUO know the content of their drugs might reduce overdose risk. Fentanyl test strips, which allow PWUO to test their drugs for the presence of fentanyl, have been found in community studies to reduce consumption to some extent, although willingness to dispose of drugs was not supported (78, 127). Further research is needed to establish the impacts of these testing capabilities, particularly in light of growing fentanyl pervasiveness.

### Demand-Side Measures: Expanding Treatment for Opioid Use Disorder

A central component of policy and practice responses to the opioid epidemic has been to increase the availability of and access to evidence-based treatments for OUD. There are three FDA-approved medications for opioid use disorder (MOUD): methadone, buprenorphine, and long-acting naltrexone. While all three are effective at reducing opioid use among people with OUD (98), the opioid agonist medications methadone and buprenorphine have the longest-standing evidence base and are considered the gold standard of care for OUD (32). Multiple longitudinal studies reveal the protective effect of MOUD against overdose risk among PWUO relative to periods with no treatment (94) or treatment with nonmedication behavioral therapies (91).

Despite the strong scientific evidence base for MOUD in reducing opioid-related morbidity and mortality among PWUO, large gaps in access to these treatments remain (161). The majority of persons who enter treatment for OUD across the United States do not receive medication (89), and most substance use treatment programs do not offer MOUD as an option (111). Furthermore, of the minority of patients who do access MOUD (5), most discontinue treatment within the first few weeks or months (115, 147). Risk of overdose increases substantially in the first few weeks following cessation (161). Thus, more research is needed to understand the population-level impact on mortality of current efforts to increase access to MOUD.

Multiple structural, social, and behavioral factors contribute to low utilization and retention of MOUD. For one, regulatory and financial hurdles related to the medical infrastructure and staffing required for the provision of controlled medications often act as barriers to incorporate MOUD into existing treatment programs (84). Methadone can only be dispensed through specialty treatment settings licensed by the Drug Enforcement Agency (DEA), and it requires daily in-person visits from patients. Buprenorphine can be prescribed as a take-home medication, but only by physicians who obtain a special waiver from the DEA. These requirements have led to a dearth of treatment programs and providers that have the infrastructure and training to prescribe medications, especially in more remote areas that have not historically seen high rates of opioid addiction (76, 142). In addition, MOUD programs often have stringent requirements such as frequent visits, mandatory participation in adjunct behavioral services, or abstinence conditions that can become burdensome or unattainable for many patients (72).

Persistent stigma and ideological resistance to medication treatment further contribute to low MOUD treatment engagement and retention, as well as to difficulty in expanding the availability of programs that offer MOUD (156). The widespread philosophy of abstinence still presides over much of the substance use treatment community, with the misunderstanding that MOUD merely replace one addiction for another (75). Many persons who participate in 12-step groups as part of their recovery, for example, have described feeling stigmatized for using medications and pressured to come off of them (112). The stigma against medications and drug use overall is also highly prevalent among medical providers, many of whom choose not to seek a buprenorphine waiver or treat OUD due to notions that such patients are too complex or time consuming to take on (59, 70). All these barriers to treatment are further exacerbated by the chronic, relapsing nature of addiction and by patient ambivalence about treatment, which already make it difficult for patients to engage and remain in care (88).

While gaps in access to MOUD are substantial overall, certain groups are especially lacking in access to effective treatments. Persons in the criminal justice system, despite being at high risk for overdose death (110), have historically had very low access to MOUD. Methadone and buprenorphine are rarely available in prisons and detention centers (6), and less than 5% of persons referred to OUD treatment in community-based settings by the criminal justice system receive medications as part of their care (92). Resistance by the criminal justice system to offer medication treatments is largely driven by stigma and misconceptions about effectiveness as well as concerns about diversion and misuse (51). Evidence about the effectiveness of MOUD in reducing overdose risk in places like Rhode Island have sparked recent efforts to expand access to MOUD, but implementation remains an ongoing challenge (51). Another group with very low access to MOUD are youth. Studies have shown that only 2% of adolescents in treatment for heroin use disorder receive medications as part of treatment (47) and that adolescents and young adults are rarely referred to MOUD following a nonfatal overdose (3). Lack of training among pediatricians, limited insurance coverage and limited availability of medications in treatment programs that serve youth, and ongoing preferences for nonmedication treatments all contribute to the low utilization rates of these medications among this group (2). Changes applied to treatment regulations in response to COVID-19 have offered an opportunity to reconsider some of the stringent requirements surrounding MOUD and to address existing barriers to make treatment more flexible and accessible (90). Ongoing research is therefore needed to assess these changes and understand their longer-term sustainability and impact on treatment access and retention (17).

## LOOKING AHEAD: PRIORITIES FOR RESEARCH

The overdose epidemic continues to evolve, with an increasing involvement of stimulants such as cocaine and methamphetamines in opioid overdoses starting in 2015 and a decline in opioid overdose deaths starting in 2018. How the overdose epidemic will develop in light of the COVID-19 pandemic remains unclear (13, 74).

Current research points to multiple social and behavioral forces on the supply and demand sides that combine to drive the different phases of the overdose epidemic. A shift in opioid prescribing practices pushed by the pharmaceutical industry and changes in norms around the treatment of chronic noncancer pain, combined with deindustrialization, reduction in work opportunities, and the targeting of people with work-related injuries and disability for high-risk opioid prescribing all likely contributed to the rise in nonmedical PO use, OUD, and overdoses. The introduction of higher-purity, lower-price heroin and the restriction of the PO supply by the enactment of new measures such as pain management clinic laws and prescription drug monitoring programs may have then contributed to a transition to heroin use among people with a prior history of nonmedical PO use. Finally, the shift in the illegal drug market to combine heroin with synthetics such as fentanyl caused the rise in overdoses involving synthetics.

Throughout the overdose epidemic, the criminalization of opioids and the incarceration of PWUO have created a group that is particularly vulnerable to complications from opioid misuse, including high risk of overdose upon release from correctional settings. Harm reduction measures such as access to naloxone and Good Samaritan laws, as well as access to treatment for OUD, are critical policy and practice levers to address the overdose epidemic. At the same time, financial and regulatory hurdles, as well as the stigma associated with opioid use and the use of medications to treat OUD, have substantially limited access to harm reduction and treatment for OUD, likely contributing to persistently high levels of overdose.

Our understanding of the social and behavioral contributors to the overdose epidemic has expanded in the past 15 years. At the same time, important directions for future research deserve mention. First, there is some evidence that access to the opioid supply, social and economic factors that drive demand, and policy measures taken to curb the overdose epidemic may differentially affect opioid misuse across racial/ethnic, gender, and urban/rural subgroups (see, e.g., 146). Such heterogeneity could be due to differences in access to opioids (in this mechanism, demand- and supply-side drivers are intertwined) (40) and/or in how demand drivers are experienced (40, 146). Examining the distinct impacts of social and behavioral drivers of the overdose epidemic on demographic subgroups should be a priority for future research. Second, the treatment of chronic pain conditions continues to be a significant challenge, and concern exists that reductions in PO access could limit access to these medications for people who need them, exacerbating pain conditions. Research into the unintended consequences of PO policies on access to pain treatment should be a priority. Third, the bulk of current research on the social and behavioral drivers of opioid misuse has focused on understanding the roots of PO misuse, particularly among Whites in deindustrialized areas. However, much less is understood about the sources of demand for heroin and synthetics and about the emerging problem of polydrug use, particularly in urban areas and among racial/ethnic minority populations. Fourth, social barriers such as the stigmatization of PWUO and institutional and financial constraints are likely shaping the extent to which local jurisdictions effectively deliver harm reduction and evidence-based OUD treatment services. Investigation into the role of stigma, local laws and policies, and the individual interests and needs of PWUO in shaping access to harm reduction and treatment, as well as research on how to reduce such barriers, should complement the substantial investment in state-level opioid policies.
